# Multidisciplinary tumor board for head and neck cancer from the perspective of medical oncologists—optimizing its effectiveness

**DOI:** 10.3389/fonc.2023.1257853

**Published:** 2023-08-30

**Authors:** Tomoya Yokota, Takashi Mukaigawa, Yoshichika Yasunaga, Hirofumi Ogawa, Tsuyoshi Onoe, Takashi Yurikusa, Aiko Yamashita

**Affiliations:** ^1^ Division of Gastrointestinal Oncology, Shizuoka Cancer Center, Shizuoka, Japan; ^2^ Division of Head and Neck Surgery, Shizuoka Cancer Center, Shizuoka, Japan; ^3^ Division of Plastic and Reconstructive Surgery, Shizuoka Cancer Center, Shizuoka, Japan; ^4^ Division of Radiation Oncology, Shizuoka Cancer Center, Shizuoka, Japan; ^5^ Division of Dentistry and Oral Surgery, Shizuoka Cancer Center, Shizuoka, Japan; ^6^ Division of Nutrition, Shizuoka Cancer Center, Shizuoka, Japan

**Keywords:** head and neck oncology, medical oncologists, multidisciplinary intervention, multidisciplinary tumor board, evidence-based medicine, personalized medicine

## Abstract

Head and neck cancer (HNC) treatment is becoming increasingly multidisciplinary, and patient characteristics vary. Therefore, a multidisciplinary tumor board (MTB) is essential in clinical practice. This review provides insights into the benefits and tips for improving head and neck MTB from the perspective of medical oncologists. The MTB is a platform to discuss the optimal application of the standard of care to each case, reach a consensus, and establish a recommendation to support patients’ decision-making. A productive and educational MTB also provides an opportunity to share information on ongoing clinical trials with physicians. Case presentations should be systematic to discuss all new and challenging cases before, during, and after the treatment. Human resource development, particularly of head and neck medical oncologists, is crucial. The type of multidisciplinary network between medical staff and the extent of patient intervention differs among MTB teams. Subsequently, a virtual MTB can establish a medical network between institutions that will contribute to the equalization and centralization of head and neck oncologic care.

## 1 Introduction

Treatment targets and strategies for head and neck cancer (HNC) are becoming more diversified and complicated. Indeed, clinicians need to consider the general condition, tumor staging, comorbidities, current and previous therapies, and patient preferences to ensure optimal cancer care for each patient. Therefore, a multidisciplinary approach is crucial in HNC care.

The National Cancer Institute (NCI) defines a multidisciplinary tumor board (MTB) as a treatment planning approach in which a group of health-care professionals, who are experts in different specialties, review and discuss the medical condition and treatment options of patients (1). MTB are now conducted worldwide for the management of patients with various cancers. A review by Fleissig et al. reported the effectiveness of MTB in terms of better team dynamics, communication, and educational opportunities for health care professionals, improved patient satisfaction, and improved clinical outcomes for patients considered by MTB versus individual care (2). Furthermore, a study revealed that a review by MTB at an NCI-designated cancer center has a diagnostic impact for many patients with breast cancer (3).

Clinical practice in HNC may differ by country owing to the reimbursement system, socioeconomic situation, and culture. For instance, HNC practice in Japan has long been led by otorhinolaryngologists, head and neck surgeons, and oral and maxillofacial surgeons. Japanese physicians hesitated to extrapolate evidence from Western countries to their practice, particularly in HNC pharmacotherapy. Since the 2000s, pharmacotherapy has been recognized as an independent subspecialty of cancer treatment in Japan, owing to its complexity and evolution. With the increasing need for knowledgeable and experienced HNC medical oncologists, multidisciplinary approach through MTB has been considered best practice in the care of HNC.

Here, we reviewed the benefits of MTB in the clinical practice of HNC from the perspective of medical oncologists. We then discussed suggestions for implementing a productive MTB. Finally, we addressed MTB concerns that require improvement and future directions.

## 2 The importance of MTB in the clinical practice of HNC

A recent meta-analysis demonstrated that the MTB improved cancer evaluation processes and survival across multiple subtypes (4). Notably, one study demonstrated that treating squamous cell carcinoma of the head and neck (SCCHN) *via* a multidisciplinary team improved survival (5). Furthermore, the Spanish Society for Head and Neck Cancer elaborated expert consensus on the multidisciplinary approach for SCCHN, and concluded that MTB is essential for achieving the best results, not only in terms of outcome, but also in terms of organ-function preservation and quality of life (6, 7).

HNC treatment is more multidisciplinary than other malignancies, because managing patients with locally advanced, recurrent, or metastatic HNC is complex. For successful HNC treatment, close cooperation among medical staff is necessary for supportive care of mucositis, skin toxicity, and nutritional support in CRT management. Various specialties provide supportive care for individual patients; thus, MTB offers an opportunity to share patient information among medical staff. Furthermore, expertise is required for its management. For instance, the development of minimally invasive surgical techniques, including transoral laser microsurgery and transoral robotic surgery (TORS), has resulted in surgery being the primary treatment for oropharyngeal cancer (8). Currently, intensity-modulated radiotherapy is more frequently used than three-dimensional conformal radiation for definitive and postoperative CRT. Proton beam and boron neutron capture therapies have also been introduced into clinical practice for HNC (9). Near-infrared photoimmunotherapy targets the EGFR and is a novel cancer phototherapy molecule (10). Furthermore, molecular targeting agents such as anti-EGFR antibodies, immune checkpoint inhibitors, tyrosine kinase inhibitors (TKIs), and classical cytotoxic agents are available for HNC treatment. In practice, it is challenging for recent clinicians to make therapeutic decisions and manage patients within an organ-specific team. Thus, MTB discussion is crucial in assessing the indications for each treatment modality and making a consensus decision.

HNC prevalence in geriatric patients is increasing (11–13), and most cases are associated with heavy smoking and drinking habits. Therefore, patients with HNC are often diagnosed with cardiovascular or cerebrovascular diseases, chronic obstructive pulmonary disease, diabetes, and renal impairment, which reduces their performance status. In MTB discussions, patient comorbidities and disease characteristics should guide the preferred treatment option.

Taken together, MTB is the best setting for such medical staff interactions.

## 3 Composition of HNC-MTB member

HNC-MTB membership varies depending on the institution. Specialists in treatment modalities, such as head and neck surgeons, otorhinolaryngologists, radiation oncologists, plastic surgeons, and medical oncologists, primarily comprise MTBs. Advice from diagnostic radiologists and pathologists helped us with the initial staging, histopathological diagnosis, and histological examination of the surgical specimens. In cases of skull base surgery, eye tumors, and malignant melanoma of the head and neck, neurosurgeons, ophthalmologists, and dermatologists may be included in the MTB. In addition to medical doctors, MTB membership is frequently expanded to include dentists, dental hygienists, physical therapists, dieticians, nurses, pharmacists, and social workers who provide supportive care. Furthermore, medical students’ participation should be encouraged because participating in the MTB is an oncology practice useful for their education. Head and neck surgeons of organ-specific divisions are often selected as the chairperson in the MTB. However, rotation may be considered.

## 4 MTB benefits from the perspective of medical oncologists

### 4.1 Establishing collaboration among medical staff in multidisciplinary cancer treatment

MTB helps to identify high-risk patients after surgery and to discuss on indications for postoperative CRT, induction or neoadjuvant chemotherapy, and optimal supportive care approaches to reduce treatment-related morbidity (14). Advanced tongue cancer treatment is an example of a multidisciplinary approach with the collaboration of medical oncologists, radiation oncologists, radiologists, head and neck surgeons, and reconstructive surgeons in HNC. This synergy enables the prompt development of an effective treatment plan for each patient in a series of glossectomies, tongue reconstruction, percutaneous endoscopic gastrostomy, and postoperative CRT (15). Furthermore, dieticians, physical therapists, dentists, and dental hygienists provide nutritional support (16, 17), rehabilitation of chewing ability and oral intake, oral care, and follow-up of radiotherapy-related toxicities, such as osteoradionecrosis, to maintain patients’ quality of life (QOL).

Medical oncologists are general physicians in cancer care who communicate closely with patients and their families. Medical oncologists are pivotal, particularly in HNC pharmacotherapy; however, they should also consider local and systemic therapies in multimodal combination and sequencing (18). Consequently, they need to be able to negotiate with other specialists as coordinators appropriately. For instance, surgical resection or palliative radiation may be required to manage locoregional diseases, even during palliative chemotherapy for recurrent or metastatic (RM)-SCCHN. In head and neck emergencies, such as tumor bleeding, infection, and airway obstruction, early referral to a head and neck surgeon is recommended. Thus, head and neck medical oncologists should always consider diverse treatment strategies.

Esophageal cancer and head and neck cancers are frequently observed simultaneously (19–21); however, their treatment strategies are often complex and challenging. The MTB, in which gastrointestinal oncologists participate, is ideal for discussing how to approach each cancer—simultaneously or sequentially. The treatment strategies include synchronous resection of both cancers, synchronous CRT for both cancers, staged resection and CRT (22, 23), or induction chemotherapy for each cancer (24). These options were selected per case based on tumor staging, invasiveness, complications, curability, and QOL, such as swallowing function. Treating multiple synchronous cancers allows medical oncologists to demonstrate their tumor-agnostic treatment skills.

### 4.2 Improvement of pharmacotherapy quality―checking the complex and diverse pharmacotherapy system

Pharmacotherapy is an important treatment modality for patients with HNC. CDDP is essential in HNC treatment, and CDDP-based concurrent CRT confers a survival benefit and laryngeal preservation in locally advanced (LA) SCCHN over radiotherapy alone (25). Treatment with cetuximab and immune checkpoint inhibitors improves the prognosis of patients with RM-SCCHN. Multitarget and selective TKIs are used for treating unresectable thyroid cancer (26–28). Thus, medical oncologists play roles in determining pharmacotherapy indications and fully and safely utilizing these agents.

Since HNC patients are often geriatrics and typically have several comorbidities, standard therapy is applied for a limited number of patients in real-world clinical practices. For instance, CDDP administration is associated with toxicities and serious adverse events in elderly patients or those with cardiac, renal, or neurogenic dysfunction. Therefore, surgeons and radiation oncologists often select radiotherapy alone for patients with LA-SCCHN. With effective communication among medical oncologists, surgeons, and radiation oncologists, MTB members may propose alternative treatment options to reduce or prevent the toxicity of high-dose CDDP-based CRT, including CDDP dose modification, modified administration scheduling, or use of alternative drugs based on individual organ function (29).

Notably, personalized treatment strategies should be proposed based on the risk-benefit ratio of each treatment option for patients ineligible for standard care. The following challenges may be discussed by the MTB for patients for whom the optimal standard care is unsuitable (**Table 1**):

1) Definitive or postoperative CRT for patients for whom CDDP is unsuitable.
2) Induction chemotherapy for patients with LA-SCCHN with high-risk disease or those for whom organ preservation is the goal but are ineligible for the docetaxel plus CDDP and 5-fluorouracil regimen.

**Table 1:** Issues to be discussed in head and neck MTB.

Tumor types	Treatment setting	Topics
SCCHN, resectable	Curative setting	Choice of upfront surgery or non-surgical treatment
LASCCHN	Definitive RT or CRT	Radiation dose, fraction, field Alternatives to definitive CRT regimen in CDDP-ineligible patients
LASCCHN, high-risk stage II laryngeal cancer	Definitive RT or CRT	Choice of RT alone or CRT
LASCCHN	ICT	Indication and purpose of ICT Alternatives to the ICT-TPF regimen
LASCCHN	Post-definitive RT/CRT	Diagnosis of post-definitive RT/CRT and its management Indication for salvage surgery
LASCCHN, oral cancer	Neoadjuvant chemotherapy	Indication and purpose of neoadjuvant chemotherapy
LASCCHN, pharyngeal/laryngeal/oral cancer, and others	Surgery and reconstruction	Surgical technique—such as setting the resection margin and reconstruction
LASCCHN, nasal and paranasal sinus cancer	Skull base surgery	Surgical technique, operation workflow
Postoperative high-risk SCCHN	Postoperative CRT	Choice of RT alone or CRT Alternatives to postoperative CRT regimen in CDDP-ineligible patients
Recurrent or metastatic disease	Palliative pharmacotherapy	Indication for pharmacotherapy Treatment regimen Indication for CGP test in rare cancer
	Palliative RT	Indication for re-irradiation Indication for stereotactic radiosurgery
Unresectable thyroid cancers	Palliative pharmacotherapy	Indication and timing of initiation of TKIs Indication for CGP test
All	Definitive and palliative setting	Symptomatic management Nutritional management Management for acute and late treatment-related toxicities Functional assessment Psychological and socioeconomic issues

SCCHN, squamous cell carcinoma of the head and neck; LA, locally advanced; RT, radiotherapy; CRT, chemoradiotherapy; CDDP, cisplatin; ICT, induction chemotherapy; TPF, Docetaxel plus CDDP and 5-fluorouracil; CGP, comprehensive cancer genomic profiling; TKI, tyrosine kinase inhibitor.

### 4.3 Establishing a consensus to support patients’ decision-making

Some patients with HNC need support in decision-making regarding treatment modalities and nutritional support. For instance, CRT is preferred for young patients with LA-SCCHN who wish to preserve their organs; however, total laryngectomy is often performed in elderly patients at high risk of aspiration pneumonia induced by definitive CRT. The patient can decide whether to undergo laryngectomy or CRT; however, medical support is essential for decision-making directly related to survival outcomes and QOL, such as eating, swallowing, and voice functions. Rather than always leaving the choice of treatment to the patient and family, establishing a consensus on the recommended treatment by the MTB and guiding the patient in decision-making are fundamental.

### 4.4 Sharing information on ongoing clinical trials

High-volume centers are often invited to company- and physician-initiated clinical trials in head and neck oncology. These institutions are responsible for participating in clinical trials. Head and neck surgeons and otorhinolaryngologists often make primary contact with new patients with HNC. The MTB shares information with these divisions on ongoing clinical trials and announces the recruitment of candidates regularly.

## 5 Tips for implementing a productive MTB

### 5.1 To optimally present all new cases

All new patients should be presented and examined by multidisciplinary specialists on the MTB, regardless of planning their initial treatment strategies, such as upfront surgery, radiotherapy, or endoscopic resection, for early-stage cancer because alternative treatment options may be proposed. The approval in the MTB should be documented.

Cases should be sequentially presented based on the categorization from the perspective of each medical department. Thus, all cases can be systematically included in the agenda. The categorization may include the following examples (**Table 1**):

1) For cases mainly treated with surgery with or without reconstruction, surgical techniques, such as setting the resection margin and reconstruction, are discussed among surgeons. Neurosurgeons and ophthalmologists also participate in discussions on skull base surgery for nasal and paranasal sinus cancers.
2) The dose, fraction, field, and palliative or definitive settings are determined for cases primarily treated with radiation. Indications for stereotactic radiosurgery of metastatic lung lesions and re-irradiation are also discussed.
3) New cases that require multimodal treatment.
4) Challenging cases during or after treatment (Section 4.2)

Head and neck medical oncologists should have the following discussions (**Table 1**).

1) Upfront surgery or non-surgical treatment in resectable laryngeal and pharyngeal cancers
2) Indications for induction chemotherapy before CRT and its purpose, such as survival improvement with a distant control and laryngeal preservation
3) Indication for neoadjuvant chemotherapy before surgery for oral cancer (30)
4) Definitive radiotherapy alone or CRT for high-risk stage II laryngeal cancer (31)
5) Adjuvant CRT or radiotherapy alone for postoperative high-risk SCCHN
6) Pharmacotherapy indication for recurrent and metastatic disease
7) Risks and benefits of re-irradiation for recurrent diseases
8) TKI initiation time for thyroid cancer
9) Indication for a comprehensive genomic profiling test for rare cancer

### 5.2 To discuss challenging cases during or after treatment

In addition to all new HNC cases, prompt information sharing on challenging cases within the MTB is necessary during or after treatment with surgery, radiation, or chemotherapy (**Table 1**). The patients tolerate the standard of care; however, the subsequent treatment course for each individual varies. Therefore, irregular adverse events may occur during the treatment.

For instance, the MTB can reach a consensus on posttreatment diagnosis and management after definitive CRT, enabling us to perform additional diagnostic modalities, such as free needle biopsy, positron emission tomography, or observation. Medical oncologists find it challenging to resolve anatomical and radiological diagnostic issues; therefore, asking head and neck surgeons and diagnostic radiologists for their opinions on MTB helps. Furthermore, determining the indications for salvage surgery for residual disease after CRT is possible. MTB can also confirm whether patients with RM-SCCHN have indications for palliative RT aimed at locoregional control (**Table 1**).

### 5.3 Discussion on an individual case basis using evidence

Standards of care and clinical practice guidelines are established based on evidence from clinical trial data. Therefore, determining a treatment plan for patients without these factors is impossible. First, all physicians involved in treating HNC should understand the updated guidelines.

However, MTB is responsible for discussing the preferred treatment strategy on an individual case basis, using evidence and guidelines. The National Comprehensive Cancer Network guidelines provide recommendations for the appropriate care of approximately 95% of patients (32). However, administering only standard treatment to each case is not feasible. Physicians should recognize that the patient characteristics in clinical trials do not completely reflect those in clinical practice. Most patients with HNC cannot be completely treated according to guidelines alone owing to various factors such as organ dysfunction, comorbidities, multiple cancers, and socioeconomic issues such as alcohol dependence, living without relatives, and being on welfare. Unfortunately, these patients are often declared untreatable and treated out of pocket because of the unavailability of standard care or a lack of evidence. Ironically, this may be the disadvantage of guideline supremacy. Evidence derived from clinical trials and standard treatments is essential; however, sufficient evidence to manage all patients with HNC with varying pathophysiology is not available. Therefore, individual patient conditions should be considered in MTB when applying these recommendations. Furthermore, patients’ requests to their healthcare providers should be provided according to their diverse values.

Thus, the MTB is a forum for discussing the appropriate assessment and response to each patient’s condition based on their physical and social needs rather than solely relying on evidence (33).

### 5.4 To create a relaxed atmosphere in MTB

MTB educates medical students, residents, and fellowship-trained young doctors; thus, they should regularly present cases and actively exchange opinions from the standpoint of their respective specialties. However, because medical staff with different positions and occupations gather at the MTB, young doctors hesitate to express their opinions. Therefore, creating a relaxed atmosphere where participants can freely speak on various issues may create a high-quality democratic MTB.

## 6 Issues to be solved in HNC multidisciplinary team

Human resource development is critical. Recently, medical oncologists with backgrounds in head and neck surgery and otorhinolaryngology have been trained. However, the number of head and neck medical oncologists remains small, and a large regional disparity exists. HNC is a highly specialized field; however, many aspects are to be learned from other fields, such as gastrointestinal and respiratory oncology. Therefore, organ-agnostic training programs for head and neck medical oncologists should be promoted in university hospitals and cancer centers.

**Figure 1:**
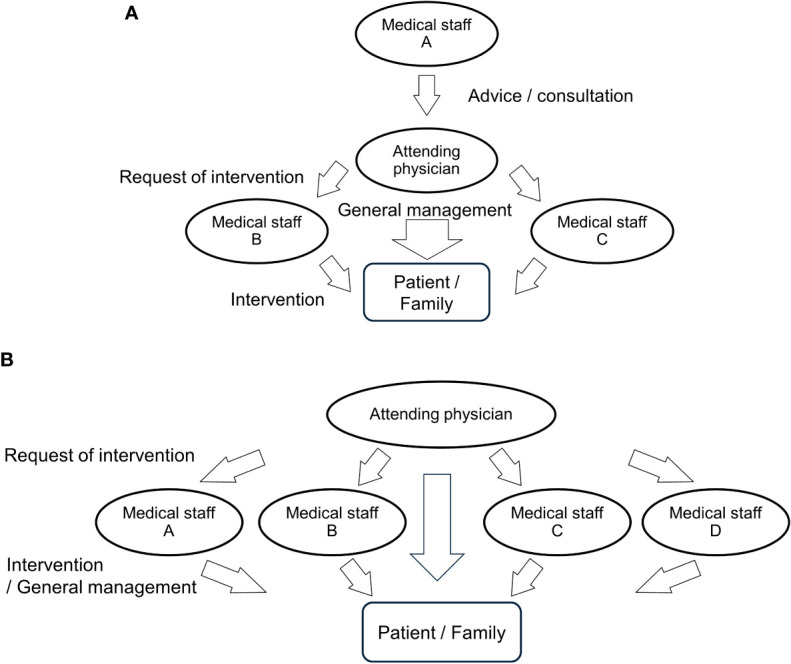
Type of multidisciplinary network among medical staff. The type of multidisciplinary network among medical staff and the extent of patient intervention differ among MTB teams. **(A)** Attending physicians are in charge of general management and communicate closely with patients. Staff A provides the attending physician with advice but lacks direct contact with patients. Medical staff B and C work on their treatment modalities at the request of the attending physician; however, they are not as involved in general management as attending physicians. **(B)** All staff members are involved in patient management, including treatment modalities and general management.

Attending physicians are central to patient management as leading physicians (**Figure 1**). The attending physician for patients undergoing non-surgical treatment in the MTB team may vary depending on the institution and region. Medical oncologists are involved in non-surgical treatment as attending physicians in the EU, the USA, and high-volume centers in Japan. Head and neck surgeons, otorhinolaryngologists, and radiation oncologists are in general hospitals in Asia-Pacific countries/regions because of the limited number of head and neck medical oncologists.

Non-attending physicians in MTBs tend to focus only on the treatment modalities of their specialties, such as radiation therapy, pharmacotherapy, reconstruction, and rehabilitation. Thus, they are undertaking only one part of the multimodal treatments. However, the attending physician oversees various patients’ management for general medical care and supportive and socioeconomical care (**Figure 1**). For instance, in treating CRT, the attending physician is involved in obtaining informed consent, managing systemic care, administering all medications, observing acute and late radiotherapy-related adverse events, emergency hospitalization, medical insurance documentation, and communication with a home doctor. However, all these responsibilities are burdensome for one physician. Approximately 56% of oncologists report an episode of emotional stress in caring for cancer patients, known as burnout, at some stage of their careers (34).

Therefore, all physicians in the MTB should view patients holistically and be proactively involved in systemic management in treating their patients. One of the solutions in the limited human resources may be to rotate attending physician among the medical departments. By doing so, it would be possible to avoid concentrating the burden of patient management on a particular department. If physicians follow each other in a multidisciplinary team and promote specialization, division, and efficiency of labor, a specific department or staff members will not be exhausted, and the resultant mental relaxation of the staff will positively affect patients and their families.

## 7 Conclusions and future direction of the head and neck MTB

The treatment strategy for HNC is becoming more complex and multidisciplinary, and patient characteristics vary; therefore, MTB is indispensable in clinically treating HNC. The MTB discusses the optimal application of standard care on an individual case basis, through which a consensus MTB recommendation is established to support patients’ decision-making. Additionally, MTB is educational, and case presentations should be systematic.

Having faced difficulties with limited clinical resources and healthcare office availability during the COVID-19 pandemic, head and neck care coordination has changed substantially. MTB has transitioned into a remote and virtual format (35–37). Virtual communication platforms will enable the implementation of MTB within large academic medical centers and multiple satellite hospitals in the future. Virtual MTB also contributes to establishing a medical network in regions of low resource availability, enhancing decentralization of head and neck oncologic care.

## Author contributions

ToY: Conceptualization, Writing – original draft, Writing – review & editing. TM: Conceptualization, Writing – review & editing. YY: Conceptualization, Writing – review & editing. HO: Conceptualization, Writing – review & editing. TO: Conceptualization, Writing – review & editing. TaY: Conceptualization, Writing – review & editing. AY: Conceptualization, Writing – review & editing.
